# Characterisation of Urine-Derived Cells for the Molecular Diagnosis of Rare Disorders

**DOI:** 10.3390/ijms27072929

**Published:** 2026-03-24

**Authors:** Karissa Ludwig, Zenghui Wu, Ghalib Bardai, Juliana Marulanda, Craig F. Munns, Pierre Moffatt, Frank Rauch

**Affiliations:** 1 Shriners Hospitals for Children—Canada, Montreal, QC H4A 0A9, Canada; 2 Child Health Research Centre, Faculty of Medicine, University of Queensland, Brisbane, QLD 4101, Australia; 3 Department of Endocrinology and Diabetes, Queensland Children’s Hospital, Brisbane, QLD 4101, Australia; 4 Faculty of Dental Medicine and Oral Sciences, McGill University, Montreal, QC H3A 2B3, Canada; 5 Department of Pediatrics, Faculty of Medicine and Health Sciences, McGill University, Montreal, QC H3A 2B3, Canada

**Keywords:** cell culture, genetic diagnosis, RNA sequencing, stem cell, urine-derived cells, urinary stem cells

## Abstract

Cultured urine-derived cells (UDCs) have been proposed as a source of material for the RNA-based molecular diagnosis of genetic disorders. Previous studies have shown that UDCs can be clonally expanded, passaged, frozen, regrown and have some stem cell characteristics, but their anatomic origin and diagnostic utility remain insufficiently explored. In this study, we cultured UDCs from 40 individuals (aged 4 to 20 years; 21 females) and extracted RNA for sequencing. We compared UDC gene expression to that of marker genes of the kidney and urinary tract segments. UDC gene expression most closely matched marker genes of parietal epithelial cells that line the inner surface of Bowman’s capsule in the kidney glomerulus. UDCs expressed *VCAM1* (CD106) and *POUF51* (OCT4), consistent with a progenitor cell type. UDCs also expressed 54.4% of 3125 OMIM-listed disease-causing genes. This indicated that UDCs can be used to diagnose a similar number of genetic disorders as skin fibroblasts and a wider range of genetic disorders than can be analysed by RNA extracted from whole blood. In conclusion, UDCs are a non-invasive cell source for RNA sequencing that is suitable for investigating a broad range of conditions.

## 1. Introduction

Urine-derived cells (UDCs) are a small subset of cells in urine samples that can be cultured, passaged, frozen and regrown and have stem cell characteristics [1]. UDCs have been used for RNA-based molecular diagnosis of various genetic disorders, such as Duchenne muscular dystrophy, Marfan syndrome, osteogenesis imperfecta, X-linked hypophosphatemic rickets and other rare disorders [2,3,4,5,6,7]. The fact that UDCs can be obtained non-invasively from a standard urine sample makes these cells an attractive source of material for RNA-based diagnosis, in particular in paediatrics.

While the vast majority of cells in urine are of epithelial origin (such as from the renal pelvis, ureters, bladder and urethra) and cannot be cultured, a tiny number of UDCs (<10 cells in a typical 50 mL urine sample) grow under suitable conditions [1]. Cell culture from the urine of newborns was first described in 1972 [8], but became more widely studied only after the 2012 publication of a simplified protocol [1]. Their rapid proliferation capacity, adherence to plastic tissue culture vessels, surface marker expression and differentiation potential along mesodermal lines highlight the similarity of UDCs to mesenchymal stem cells (MSCs) [9].

RNA-based diagnostics can be combined with DNA sequencing to discover the causes of rare genetic disorders [10]. In particular, RNA analyses can provide information about splice abnormalities and thereby allow the identification of variants that are difficult to find based on DNA sequencing alone, such as deep intronic variants leading to pseudoexons or exon skipping [2,3,4,5,6]. Nevertheless, RNA-based diagnostics require a tissue source in which genes of interest are expressed at levels that are adequate for the diagnostic assay [2,10,11]. Among clinically accessible tissues, skin fibroblasts are widely used for diagnostic RNA sequencing as they express a wide range of genes but require a somewhat invasive skin biopsy [11]. Blood can be obtained less invasively than fibroblasts but has a narrow gene expression profile [10].

Even though UDCs are a promising source of material for diagnostic purposes, the use of UDCs for molecular diagnosis faces several barriers. The gene expression profile of UDCs has not been delineated in detail, and it is, therefore, unclear which genes can be analysed using these cells. The anatomic origin of UDCs within the kidney or urinary tract and their stem cell characteristics are yet to be elucidated, which makes it difficult to interpret the physiological and pathophysiological role of these cells. Finally, the current standard protocol for culturing UDCs requires that urine samples be processed fresh (within 4 h) [1]. This limits the utility of UDCs in clinical practice, as it can be difficult to ship samples to a laboratory within this time frame.

In the present study, we used RNA sequencing in UDCs to address these barriers. We compared the gene expression profile of cultured UDCs to that of marker genes of kidney and urinary tract cells to determine the anatomical origin of UDCs. We assessed the expression of stem cell markers in UDCs, and we explored the expression of disease-associated genes in UDCs. We also describe a simple modification of the protocol for handling urine samples that allows for a longer preservation of UDCs in unprocessed urine samples.

## 2. Results

### 2.1. UDC Localisation and Characterisation

#### 2.1.1. Localisation of UDC Origin Within the Kidney (Figure 1)

UDCs were mostly negative for markers of cells lining the renal tubular system, collecting duct and urothelium (which lines the renal pelvis, ureters, bladder and proximal urethra). In contrast, markers of parietal epithelial cells (which line the inside of Bowman’s capsule) were well expressed, but markers for podocytes and glomerular endothelial cells were not expressed.

**Figure 1:**
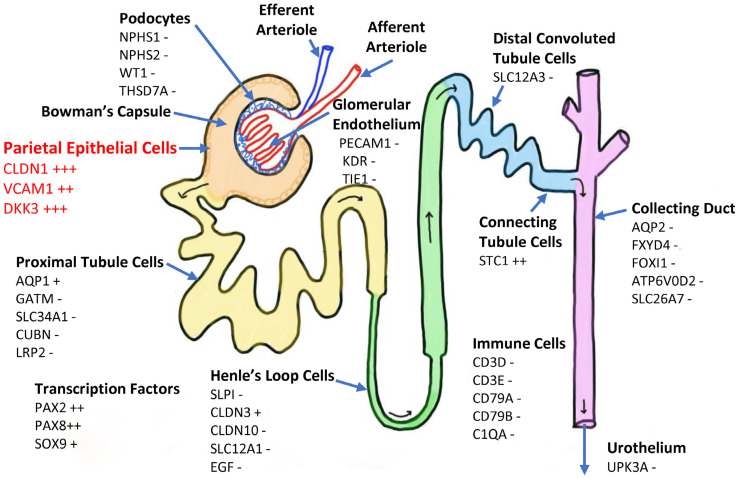
Median UDC expression levels of marker genes associated with specific regions of the kidney and urinary tract. Markers of parietal epithelial cells are highly expressed, whereas markers of kidney tubules and the urinary tract are expressed at low levels or are absent in UDCs. Gene expression: - no/very low expression (TPM < 1); + low expression (TPM 1–10); ++ moderate (TPM 10–100); +++ high (TPM 100–1000).

Several gene markers of parietal epithelial progenitor cells (*PROM1*, *PAX2*, and *SOX9*) were also expressed in UDCs [12]. UDCs expressed *VCAM1* (CD106), which is a marker of renal progenitors committed towards the tubular lineage [13]. Overall, the gene expression pattern found in UDCs is compatible with the hypothesis that UDCs have their origin in progenitor parietal epithelial cells [14,15].

#### 2.1.2. Expression of Stem Cell Marker Genes (Figure 2)

Expression of genes associated with embryonic stem cells (ESCs) was compared across tissue types. Of the 30 ESC genes that were assessed, 14 (47%) were expressed (TPM > 1) in UDCs, compared with nine (30%) in whole blood, 15 (50%) in fibroblasts and 18 (60%) in adipocytes. *POUF51* (OCT4) and *SMAD2* showed stronger expression in UDCs than in the other tissue types. *KLF4* and *MYC* showed lower expression in UDCs compared with adipocytes and fibroblasts. *NANOG* and *SOX2* were not seen in any tissue type. All four tissue types expressed a higher proportion of MSC genes than ESC genes. UDCs expressed all 10 (100%) MSC genes examined, compared with nine (90%) expressed in both fibroblasts and adipocytes, and six (60%) in whole blood. UDCs did not express any hematopoietic stem cell marker genes.

**Figure 2:**
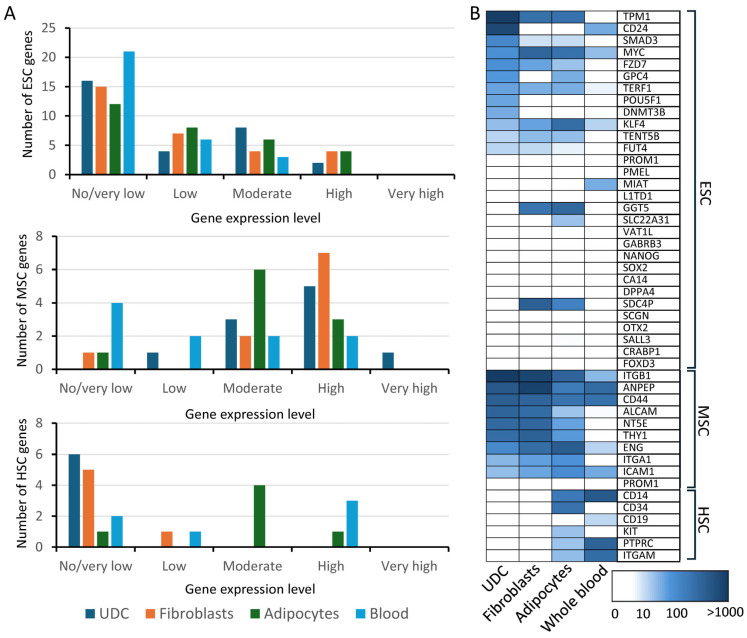
Expression of embryonic, mesenchymal and hemopoietic stem cell marker genes across tissue types: (**A**). Expression of embryonic stem cell (ESC), mesenchymal stem cell (MSC) and hemopoietic stem cell (HSC) marker genes by tissue type. No/very low expression: TPM < 1; low: TPM 1–10; moderate: TPM > 10–100; high: TPM > 100–1000; very high: TPM > 1000. (**B**). Heatmap showing expression of stem cell marker genes across tissue types. TPM = transcripts per million.

### 2.2. Utility of UDCs for RNA-Based Diagnosis

#### 2.2.1. Comparison of Gene Expression Across Clinically Accessible Tissue Types (Figure 3)

Gene expression was compared between UDCs (*n* = 40 samples, results from the present study) and three other clinically accessible tissue types as available in the GTEx database (gtexportal.org): whole blood (*n* = 755 samples), fibroblasts (*n* = 504 samples) and adipocytes (*n* = 663 samples). A principal component analysis using the results of all the expressed genes (TPM > 1) demonstrated distinct clusters of gene expression by tissue type. The principal component analysis of UDC gene expression visualised by age group, sex and RNA sequencing run demonstrated no distinct clustering of gene expression, indicating that the overall gene expression did not appear to be substantially impacted by any of these parameters.

**Figure 3:**
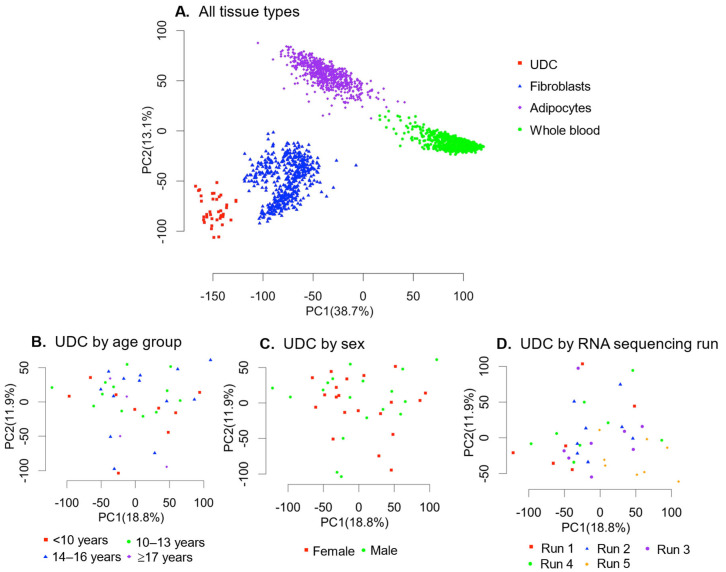
Principal component analysis of all the genes expressed (TPM > 1) in RNA sequencing data: (**A**). Gene expression of UDCs compared with GTEx data for fibroblasts, adipocytes and whole blood, indicating a distinct clustering of UDCs. (**B**–**D**). Principal component analysis displaying UDC gene expression divided by age group (**B**), sex (**C**) and RNA sequencing run/flow cell (**D**). No clustering of UDC gene expression based on any of these parameters was observed.

#### 2.2.2. Comparison of Highly Expressed Genes

Comparison of the top 10% of the expressed genes in each tissue type (2435 genes per tissue type; 4697 genes total) showed that 580 genes were expressed at high levels (TPM > 10) only in UDCs. Of these, 117 genes were listed in OMIM as disease-causing. Several of these genes showed low or no expression (TPM < 10) in any of the other tissue types analysed. These included genes were associated with homocystinuria (*CBS*; OMIM #236200), MODY-5/renal cysts and diabetes syndrome (*HNF1B*; OMIM #137920), thyroid dysgenesis (*PAX8*; OMIM #218700), and myopia with cataracts and vitreoretinal degeneration (*P3H2*; OMIM #614292).

#### 2.2.3. Expression of Clinically Relevant Genes for RNA Sequencing Analysis (Figure 4)

Next, we assessed the utility of UDCs as a source of mRNA in the diagnosis of Mendelian disorders. Genetic abnormalities can only be detected by RNA sequencing if they occur in a gene that is expressed at sufficiently high levels, defined here as TPM > 10 [2,10]. Among the 3125 OMIM-listed genes that were investigated, 1701 genes (54.4%) were expressed at a TPM > 10 in UDCs, compared with 1593 genes (51.0%) in GTEx data for fibroblasts, 1589 (50.8%) for adipocytes and 738 (23.6%) for whole blood.

**Figure 4:**
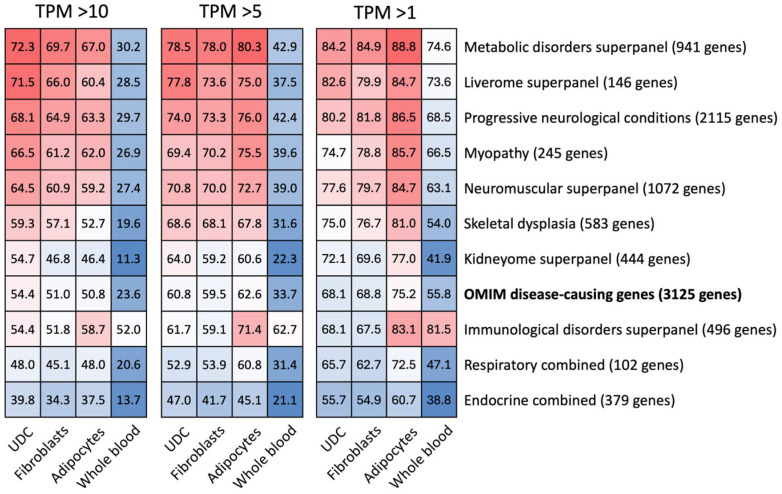
Percentage of genes in each tissue type meeting median TPM thresholds in OMIM disease-causing genes (bold) and gene lists for various disorders from PanelApp. TPM > 10 is considered sufficient expression for RNA sequencing analysis. The Respiratory combined gene list includes PanelApp panels for Interstitial lung disease, Pneumothorax, and Pulmonary fibrosis panels. Endocrine combined gene list includes PanelApp panels for Calcium and phosphate disorders, Congenital hypothyroidism, Diabetes insipidus, Hyperinsulinism, Hypertension and aldosterone disorders, Hyperthyroidism, MODY, Pituitary hormone deficiency, Primary ovarian insufficiency, and Disorders of sex differentiation panels. In each heatmap, numbers at the 50th percentile are shown in white, numbers above the 50th percentile are shown in red, numbers below the 50th percentile are shown in blue.

Additionally, we compared the gene expression from each tissue type across various gene lists from PanelApp Australia [16] to investigate the utility of UDCs for RNA sequencing analysis in different types of disorders. We found that UDCs expressed the greatest percentage of genes in 8 of the 10 gene lists analysed, with equal expression to adipocytes in a combined Respiratory disorders gene list and the second highest expression in the Immunological disorders gene list (Figure 4). The Metabolic disorders panel (72.3% of genes) and Liverome panel (71.1%) demonstrated the highest expression in UDCs compared with other panels. The gene list with the lowest expression in UDCs was Endocrine combined (39.8%), though this was still higher than the expression seen in other tissue types.

We also assessed gene expression for different TPM thresholds. As the TPM threshold lowered, the expression observed in other cell types, particularly adipocytes, increased. At median TPM > 1, adipocytes expressed 2351/3125 (75.2%) of the OMIM disease-causing gene list. The percentages expressed by UDCs and fibroblasts were similar at this TPM threshold: 2129 (68.1%) and 2150 (68.8%) genes respectively, which were still higher than expression in whole blood (1745; 55.8%). These data indicate that adipocytes would be a reasonable alternative tissue choice to explore for poorly expressed genes, even though UDCs are the only of the assessed cell types that can be obtained non-invasively.

### 2.3. Preservation Capacity for UDC Culture

The RNA sequencing results presented above used UDCs cultured from fresh urine samples, stored on ice for no longer than 4 h prior to processing according to established methodology [7]. To test a method to preserve urine samples for longer periods of time, we collected 12 urine samples (three samples each from four healthy controls). Each sample was divided, and UDC growth was compared across aliquots according to preservation method and time. UDC growth (≥1 primary colony) was observed in 11/12 aliquots processed fresh (92%). Ten of the 12 aliquots (83%, *p* = 1.00 compared to fresh sample using McNemar’s chi squared test) stored with preservation medium for 24 h prior to processing showed UDC growth, as did nine aliquots (75%, *p* = 0.48) stored without preservation medium for 24 h and eight aliquots (67%, *p* = 0.25) stored with preservation medium for 48 h.

There was no statistically significant difference in the median number of colonies grown in the fresh vs. the 24 h aliquot with preservation medium (7.5 vs. six colonies, *p* = 0.17 using the Wilcoxon signed rank test). The aliquots stored for 48 h with preservation medium and those stored for 24 h without preservation medium had a lower median yield at two colonies and one colony per aliquot respectively, which were both significantly lower than the sample processed fresh (*p* = 0.004 and *p* = 0.004).

## 3. Discussion

In this study, we cultured UDCs from 40 children, adolescents and young adults and performed RNA sequencing to characterise UDC gene expression. We found that UDCs showed high expression of genes that are specifically expressed by parietal epithelial cells in the Bowman’s capsule and included a range of MSC markers. Gene expression in UDCs was distinct from the other tissue types on principal component analysis. We demonstrated the diagnostic utility of UDCs in that a high proportion of disease-related genes were expressed at sufficiently high levels to detect abnormalities by RNA sequencing. Finally, we developed a modified protocol for the processing of urine samples that allowed preserving cells for up to 48 h prior to culture.

### 3.1. Localisation

Our analysis of the gene expression corresponding to markers specific to various cell types within the kidney showed a pattern consistent with parietal epithelial cells (lining of Bowman’s capsule) [15]. Parietal epithelial cells are thought to act as progenitor cells for podocytes and for tubular cells [13], which may explain their stem cell characteristics. Our observations are in line with a previous report that UDCs originate in the kidney rather than the urinary tract, as evidenced by a donor Y chromosome present in UDCs derived from a 46XX female renal transplant recipient [17]. Our results also indicate that UDCs are not urothelial cells, as markers for urothelial cells were negative. Even though a large number of epithelial cells, presumably of urothelial origin, is found in urine samples, such epithelial cells do not attach to the plastic cell culture vessel and are discarded with the first change in the culture medium in a newly established UDC culture.

### 3.2. Stem Cell Marker Expression

UDCs display multiple stem cell characteristics, including their adherence to plastic culture vessels, capacity for rapid expansion and ability to tolerate multiple passages [18]. Additionally, the cells have shown some capacity to directly differentiate into osteogenic, adipogenic, chondrogenic and myogenic cell lines [17,19,20,21,22,23]. Expression of various MSC and ESC markers has been documented in UDCs using flow cytometry and immunofluorescence [18,19,20,21,23,24].

Our examination of the four transcription factors typically used to induce pluripotency in somatic cells [25] found moderate expression of *POUF51* (OCT4), *KLF4* and *MYC* in UDC, whereas *SOX2* was not expressed. *POUF51* (OCT4) is highly expressed in pluripotent stem cells, and reduced expression is associated with induction of cell differentiation [26]. The moderate expression of *POUF51* (OCT4) in UDCs with low/no expression in other tissue types supports the proposition that UDCs retain some stem cell characteristics. *NANOG* is only expressed in undifferentiated cells and is essential in the conversion of pre-pluripotent cells into fully reprogrammed induced pluripotent stem cells, and therefore appears to have a key role in this differentiation step [27]. The absence of *NANOG* expression in UDCs, therefore, indicates that these cells lack pluripotency and have started down the pathway of lineage selection [26]. The MSC and ESC gene expression profile demonstrated in our UDCs potentially supports the multipotency of UDCs and may explain their observed similarities to MSCs.

### 3.3. Utility of UDCs in RNA Sequencing Analysis

UDCs demonstrated utility as a source of cells for RNA-based diagnostic analysis in both gene expression profile and capacity for sample preservation prior to culture. UDCs expressed more than half of all OMIM disease-causing genes and demonstrated similar or superior gene expression compared with other tissue types across a broad range of gene panels from PanelApp Australia at an expression threshold considered appropriate for RNA sequencing analysis (TPM > 10) [2,10]. When lower gene expression thresholds (TPM > 5 and >1) were explored, the gene expression in adipocytes and fibroblasts compared to UDCs improved. It would, therefore, be reasonable to consider these tissue types when targeting specific poorly expressed genes; however, both of these require an invasive procedure for sample collection. A challenge in the widespread application of UDC culture is the necessity to process samples within 4 h of collection [1], after which a substantial reduction in colony yield has been demonstrated [24]. Attempts at sample preservation for up to 24 h have been reported [28]; however, the preservation media used were relatively specialised and expensive, and the practice has not been widely adopted. We have demonstrated that mixing urine samples with a simple, inexpensive and readily accessible preservation medium prior to refrigeration for up to 48 h did not result in a statistically significant reduction in the number of urine samples producing at least one primary UDC colony. This simple preservation technique could facilitate urine sample collection from distant centres, with refrigerated shipping to the laboratory for UDC culture, thereby increasing the practical viability of UDCs for clinical diagnosis.

### 3.4. Limitations

We compared the UDC transcriptome to data obtained in other tissues that were analysed by the GTEx consortium. The results may therefore be influenced by methodological differences between studies. Additionally, the majority (30/40) of the participants in our study had a genetic disorder, which may have affected the expression of some of the genes involved. However, none of the participants had a disorder with anticipated effects on renal function or expression of genes within the kidney, so any impact on the localisation and general gene expression data would likely be minimal.

The gene expression data generated in this study came from a paediatric cohort, which raises the possibility of age-related differences in the gene expression profile compared with the GTEx data, which is based on an adult population. However, the principal component analysis of our UDC data did not demonstrate any clustering of overall gene expression based on age (nor sex or RNA sequencing run). This indicates that the differences observed between the UDCs and other tissue types are more likely related to differences in tissue-based gene expression rather than age.

## 4. Materials and Methods

### 4.1. Subjects

Clean-catch urine samples for RNA sequencing were collected from 40 participants (30 patients with primary bone disorders and 10 healthy controls; age range of 5 to 20 years; 21 female) at Shriners Hospitals for Children—Canada in Montreal. Urine samples were obtained from a further 4 healthy adult participants (2 female, age range 18–40 years) at the Child Health Research Centre, University of Queensland, for analysis of preservation techniques. Informed consent was obtained from a parent/legal guardian of participants younger than 18 years and from the participant if aged 18 years or older. Ethics approval for the Canadian study was obtained from the McGill University Institutional Review Board (IRB study no. A04-M10-21A). Ethics approval for the Australian study was obtained from Children’s Health Queensland Hospital and Health Service Human Research Ethics Committee (HREC/22/QCHQ/88446). Studies at both sites were conducted according to the principles of the Declaration of Helsinki.

### 4.2. Sample Preparation and RNA Sequencing

The urine samples for RNA sequencing analysis were processed as previously described [3,7]. Briefly, the urine samples were centrifuged, and the cell pellet was washed and resuspended in a primary growth medium, then plated in a 12-well plate. The medium was changed to proliferation medium on day 4, with subsequent medium changes every 2 to 3 days. Cells were passaged when the primary colonies covered approximately a quarter of the well surface. RNA for sequencing was generally extracted at passage 2 (range passage 1 to 3; culture day 12 to 28).

RNA preparation and sequencing were performed as described [3]. Briefly, RNA was extracted using the TRIzol method (ThermoFisher Scientific, Waltham, MA, USA). Libraries were generated using 100 ng of total RNA with mRNA enrichment by both poly-A selection and ribosomal RNA depletion. Libraries were prepared using the NEBNext Ultra II Directional RNA Library Prep Kit (NEB, Ipswich, MA, USA, E7645). cDNA synthesis was achieved by the NEB- Next Ultra Directional RNA First Strand Synthesis and the Second Strand Synthesis Modules. Sequencing was performed on an Illumina (Victoria, BC, Canada) NextSeq550 device using a high-throughput flow cell with eight samples multiplexed (150 bp paired-end reads). Base calling was performed with RTAv3, and bcl2fastq2 v2.20 was used to demultiplex the samples and generate fastq reads. The fastq reads quality control was evaluated by FastQC (version 0.11.9), and aggregated results were generated by MultiQC (version 1.14). Alignment to the human hg19 reference genome was performed using the STAR aligner (version 2.7.9a). Read counts were determined using Samtools (version 1.16.1).

### 4.3. Quantification of Gene Expression

StringTie (version 2.2.1) was used for the quantification of gene expression, and the gene abundance estimates were expressed as transcripts per million (TPM) [10,11]. A TPM threshold of 1 was used to distinguish ‘expressed’ from ‘not expressed’ genes, and a TPM > 10 was generally considered sufficient for the detection of transcript abnormalities by RNA sequencing analysis [2,10,11]. In this study, we divided gene expression into the following categories: no/very low expression (TPM < 1), low expression (TPM between 1 and 10), moderate expression (TPM > 10–100), high expression (TPM 100–1000) and very high expression (TPM > 1000).

### 4.4. Characterisation of UDC Origin

As UDCs originate from within the kidney [15], marker genes for various renal and urinary tract cell types were identified from the literature [12,13,29]. The median TPM for each gene from all 40 study participants was used to determine the potential cellular origin of UDC.

To explore stem cell characteristics of UDC, lists of genes expressed in embryonic, mesenchymal and hematopoietic stem cells were compiled from the literature [18,30,31] (Figure 2B). Expression of these genes (based on median TPM) was examined for UDC. To provide context, expression of the various stem cell markers was compared to the GTEx data for three other mature tissue types as outlined below.

### 4.5. Utility of UDCs for RNAseq Analysis

#### 4.5.1. Gene Expression Across Clinically Accessible Tissue Types

To compare gene expression across different accessible tissue types used as sources of RNA for diagnostic sequencing analysis, the median TPM from our UDC RNA sequencing data was compared to publicly available TPM data obtained from the GTEx portal for whole blood, fibroblasts, and adipocytes (https://www.gtexportal.org/home/datasets, accessed on 30 June 2023). These data were compared using principal component analysis (PCA) for all the genes that were expressed (TPM > 1) in at least one of the four tissue types (15,381 genes). The top 10% of expressed genes for each tissue type were compared (2435 genes per sample from the entire list of 24,359 genes), and genes unique to UDCs within this were identified.

#### 4.5.2. Expression of Clinically Relevant Genes

Next, we assessed which proportion of genes in various diagnostic gene panels had a median TPM > 10 in UDCs, whole blood, fibroblasts and adipocytes. This analysis was performed for a list of 3125 genes that are associated with Mendelian disorders in the OMIM database (Supplementary Table S1) [2,32], and for gene panels associated with various disease types that were obtained from PanelApp Australia, a publicly available database of curated gene panels [16]. We assessed the following panels: Progressive neurological conditions (version 14.832; 2115 unique genes), Immunological disorders superpanel (version 9.246; 496 genes), Neuromuscular superpanel (version 3.93; 1072 genes), Myopathy (version 4.54; 245 genes), Metabolic disorders superpanel (version 8.79; 941 genes), Liverome superpanel (version 1.3; 146 genes), Kidneyome superpanel (version 8.50; 444 genes) and Skeletal dysplasia (version 0.272; 583 genes). A Respiratory combined list (102 genes total) including the PanelApp Australia panels for Interstitial lung disease (version 1.0), Pneumothorax (version 0.11), and Pulmonary fibrosis (version 0.54) and an Endocrine combined list (379 genes total) including PanelApp Australia panels for Calcium and phosphate disorders (version 1.0), Congenital hypothyroidism (version 0.43), Diabetes insipidus (version 1.3), Hyperinsulinism (version 1.9), Hypertension and aldosterone disorders (version 1.14), Hyperthyroidism (version 0.23), Monogenic diabetes (version 0.50), Pituitary hormone deficiency (version 0.34), Primary ovarian insufficiency (version 0.323), Differences in sex development (version 0.293) were also assessed. We also assessed the percentage of genes with a median TPM > 5 and >1 to explore whether the gene expression data held across different threshold choices.

#### 4.5.3. Preservation Capacity

To further assess the utility of UDCs as a cell source for RNA sequencing analysis, the capacity to establish UDC primary colonies from preserved (rather than fresh) urine samples was investigated. A total of 12 urine samples were obtained from 4 adult healthy controls aged 18 to 40 years (3 samples each on 3 different days; volume 100 to 430 mL) at the University of Queensland Child Health Research Centre, Brisbane, Australia. The samples were each mixed well, then divided into 4 equal aliquots. A preservation medium (50:50 DMEM/Ham’s F12 with 5% FBS) was added to 2 of the aliquots at 10% *v*/*v*. One aliquot was processed fresh for UDC culture as described above. The aliquots containing preservation medium were stored at 4 °C, then processed for culture at 24 and 48 h. The remaining aliquot was stored at 4 °C without preservation medium and processed for culture for 24 h. On culture day 12, the number of primary cell colonies produced by each sample aliquot was counted as a measure of yield. The colony yield was expressed as a median [interquartile range] across all the samples, with paired comparison to baseline yield (fresh sample) using the Wilcoxon signed rank test. The presence vs. absence of any colony growth was compared to the fresh sample using McNemar’s chi-squared test for paired proportions. All the statistical analyses were performed in R (version 4.3.2).

## 5. Conclusions

UDCs are a non-invasive cell source with some stem cell characteristics. Their gene expression profile indicates that they are progenitor cells arising from the parietal epithelium lining Bowman’s capsule. Their favourable gene expression profile and capacity for sample processing up to 48 h after collection make UDCs a highly useful cell source for diagnostic RNA sequencing analysis.

## Data Availability

The original contributions presented in this study are included in the article/Supplementary Materials. Further inquiries can be directed to the corresponding author.

## Supplementary Materials

The following supporting information can be downloaded at: https://www.mdpi.com/article/10.3390/ijms27072929/s1.

## Abbreviations

The following abbreviations are used in this manuscript:

ESC	Embryonic stem cell
mRNA	Messenger RNA
MSC	Mesenchymal stem cell
PCA	Principal component analysis
TPM	Transcripts per million
UDC	Urine-derived cell
